# Associations between insulin resistance and adverse pregnancy outcomes in women with gestational diabetes mellitus: a retrospective study

**DOI:** 10.1186/s12884-021-04006-x

**Published:** 2021-07-23

**Authors:** Jing Lin, Hua Jin, Lei Chen

**Affiliations:** 1 The Second School of Clinical Medicine, Southern Medical University, Guangzhou, 510515 China; 2 Department of Obstetrics and Gynecology, The Sixth Medical Center of PLA General Hospital, Beijing, China; 3 Naval Clinical College, Anhui Medical University, Anhui, China

**Keywords:** Gestational diabetes mellitus, Insulin resistance, Pregnancy outcomes

## Abstract

**Background:**

This study aimed to explore the relationship between insulin resistance (IR) and adverse pregnancy outcomes in women with gestational diabetes mellitus (GDM), and to determine the risk factors for IR in women with GDM.

**Methods:**

This study employed a retrospective survey of 710 women diagnosed with GDM. Serum lipids, fasting plasma glucose (FPG), glycosylated hemoglobin (HbA1c), and serum protein were measured in the first trimester (6–12 weeks), and OGTT and fasting insulin tests were performed in the second trimester (24–28 weeks). These results were then used to evaluate IR by homeostasis model assessment (HOMA). When HOMA-IR ≥ 2.0, IR was diagnosed. The relationship between HOMA-IR and adverse pregnancy outcomes was analyzed by a logistic regression model, and multiple stepwise regression was used to analyze the risk factors of IR.

**Results:**

IR significantly increasd the risk of the hypertensive disorders of pregnancy and large for gestational age (LGA) (*OR* = 5.31,*95%CI*:1.87,15.10; *OR* = 1.65,*95%CI*:1.10, 2.48, respectively) in women with GDM, but not for cesarean section, premature delivery, premature rupture of membranes, postpartum hemorrhage, macrosomia and SGA. Compared to normal groups, greater body mass index (BMI) before pregnancy category (overweight or obesity group) were associated with higher risk of IR in the second trimester, the *OR (95% CI)* were 4.09 (2.65, 6.30) and 6.52 (2.99, 14.20). And higher level of FPG (*OR* = 1.63, *95%CI:* 1.11, 2.40), TG (*OR* = 1.32, *95%CI:* 1.08, 1.63) and weight gain before diagnosis of GDM (*OR* = 1.08, *95%CI:* 1.02, 1.15) were also associated with higher risk of IR in the second trimester in women with GDM, while age (*OR* = 0.94, *95%CI*: 0.90, 0.98)was the weak protective factor for IR.

**Conclusion:**

GDM with IR in the second trimester increased adverse pregnancy outcomes, especially the risk of hypertensive disorders of pregnancy and LGA. In addition, FPG, HbA1c, and TG in early pregnancy, pre-pregnant BMI and weight gain before diagnosis of GDM were all independent risk factors for IR.

## Highlights

• Increasing IR in the second trimester increases risk of hypertensive disorders of pregnancy and LGA in women with GDM.
• FPG, HbA1c, and TG in early pregnancy, pre-pregnant BMI, and weight gain before diagnosis of GDM are all independent risk factors for the development of IR in the second trimester.

## Introduction

Gestational diabetes (GDM) is a common disease during pregnancy. According to a report from the International Diabetes Federation (IDF) in 2019, 12.8% of pregnant women suffer from it worldwide [1]. In China, the incidence of GDM has reached 14.8%, with an increasing growth trend [2]. GDM can bring about some short-term and long-term disorders to women and their babies, which has attracted increasing attention from the entire society.

It is now believed that GDM is associated with insulin resistance (IR) [3]. IR is a state in which normal concentrations of insulin that the normal concentration of insulin can not elicit a response of target cells, and the negative feedback urges the body to secrete excess insulin. Previous literature show that excessive gestational weight gain (GWG)[4], overweight or obesity [5], personal history of GDM [6], family history of diabetes [6], westernized diet [7], advanced maternal age [8], intrauterine nutrition status [9], are high risk factors for IR. When insulin secretion fail to compensate for IR, GDM emerges. Physiological IR during pregnancy is beneficial to fetal growth and can effectively supply nutrients [10], but the degree of IR is significantly higher than that of normal pregnancy, which will cause many adverse effects on mother and fetus, such as preterm delivery, cesarean, macrosomia, neonatal hypoglycaemia polyhydramnios, hypertensive disorders of pregnancy, fetal growth restriction and other serious complications [11–14].

In practice, once GDM is diagnosed, individualized treatment plans involving diet control, appropriate regular exercise, and insulin intervention are routinely made immediately by doctors for patients. However, it has been found that even when the recommended plans are well followed, the pregnancy outcomes cannot be effectively improved [15]. Some researches ascribes this to IR [16] and the heterogeneity of IR in pregnant women with GDM somehow results in different outcomes [11, 17]. Therefore, more exploration on the relationship between IR in the second trimester (24–28 weeks) and adverse pregnancy outcomes in GDM women may help identify women who are at high risk of these adverse outcomes, and management can therefore be carried out in early pregnancy even pre-pregnancy, sometimes adding insulin sensitization therapy if necessary to improve the prognosis.

This study aimed to determine whether IR in the second trimester is associated with certain specific adverse pregnancy outcomes, and to further explore the risk factors of IR in in the second trimester in Chinese GDM women.

## Methods

### Material and methods

**Fig. 1:**
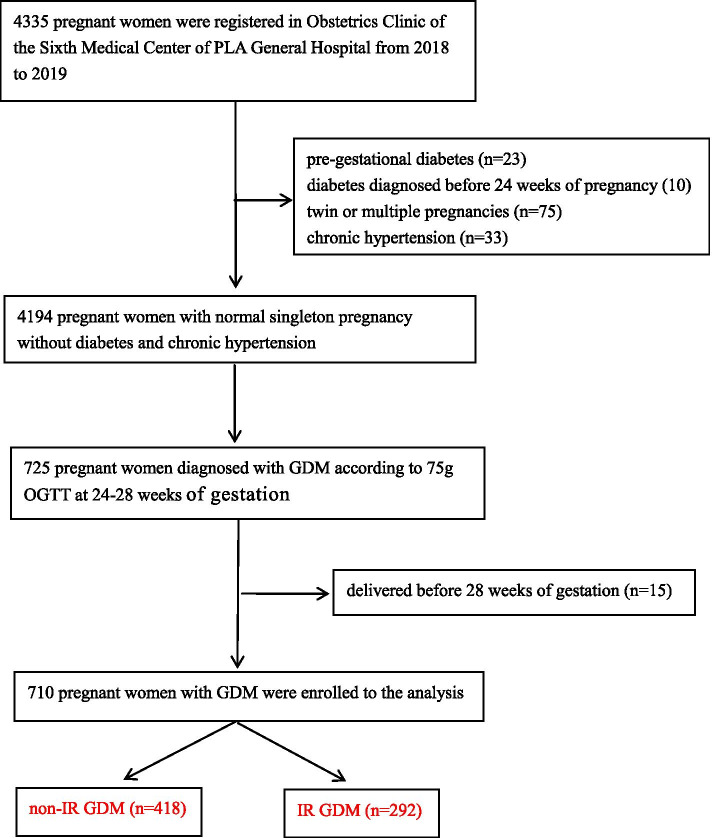
Chart for the selection of participants

This is a case–control study which included GDM women with IR (cases) and GDM women without IR (controls). And 710 GDM women were treated at the Obstetrics Clinic of the Sixth Medical Center of PLA General Hospital from January 1, 2018 to December 31, 2019 (Fig. 1). All study participants gave verbal informed consent, this was approved by the Sixth Medical Center of PLA General Hospital Ethics Committee as it was a retrospective study and no interventions was given to the participants. The study was conducted adherence to Declaration of Helsinki. All women who were not diagnosed with diabetes before 24 weeks of gestation were given 75 g oral glucose tolerance test (OGTT) at 24–28 weeks of gestation. GDM was diagnosed using the standards of the International Association of Diabetes and Pregnancy Study Group (IADPSG) [18]. The inclusion criteria were as follows: Women with pre-gestational diabetes, diabetes diagnosed before 24 weeks of pregnancy( FPG ≥ 7.0 mmol/L, HbA1c ≥ 6.5%, and/or random plasma glucose ≥ 11.1 mmol/L), twin or multiple pregnancies, chronic hypertension, and women who gave birth before 28 weeks were excluded from the study.

### GDM prenatal care

After diagnosis, all the GDM women were referred to the nutrition clinic to receive guidance on GDM diet and exercise. After 7 days of self-adjustment to their diet and activities, venous blood was drawn to check the blood sugar before and 2 h after meals, and based on the results the nurse would further adjust their diet and physical activity plans and teach them usual blood glucose monitoring at home. GDM women self-monitored the blood sugar before and 2 h after meals by a glucometer with a test strip. Target ranges for glycemic control were: fasting plasma glucose below 5.3 mmol/L, and 2 h postprandial blood glucose below 6.7 mmol/L. Generally, poor glycemic control was defined and insulin therapy is given, when blood glucose exceeds target ranges.

### General information collection

The basic information of the pregnant women was collected by an obstetrician, including the woman's age, parity, history of GDM, family history of diabetes, IVF-ET, weight before pregnancy, weight at diagnosis of GDM, weight at delivery, height, gestational week of delivery, delivery method, outcome, newborn weight, and other relevant parameters. The weight before pregnancy was measured during the pre-pregnancy check-up. If there was no pre-pregnancy check-up data, it was taken at the first pregnancy check-up. The weight at delivery was collected when they were admitted to the hospital for delivery. The weight gain before diagnosis of GDM was calculated by subtracting the weight before pregnancy from the weight at diagnosis of GDM. The total weight gain during pregnancy (GWG) was calculated by subtracting the weight before pregnancy from the weight at delivery. The newborns’ basic information was recorded immediately after birth. All the data and information related to pregnancy outcomes was extracted from electronic medical records.

### Primary outcomes

The primary outcomes of this study was adverse pregnancy outcomes, including cesarean, premature delivery, premature rupture of membranes, hypertensive disorders of pregnancy, postpartum hemorrhage, macrosomia, small for gestational age (SGA), and large for gestational age (LGA).

### Definition

**GDM:** any one of the following values was met or exceeded in 75 g OGTT: 0 h (fasting), 5.1 mmol/L;1 h, 10.0 mmol/L; and 2 h, 8.5 mmol/L. **Preterm delivery**: delivery between 28 and 37 weeks of gestation. **Premature rupture of membranes**:a spontaneous rupture of membranes that occurs before delivery. **Hypertensive disorders of pregnancy**: hypertension during pregnancy, preeclampsia, and eclampsia. **Postpartum hemorrhage**: For vaginal delivery, the bleeding volume exceeded 500 mL within 24 h after delivery of the fetus; for cesarean section the bleeding volume exceeded 1,000 ml. **Small for gestational age infant (SGA) and large for gestational age infant (LGA)**:the newborns whose birth weights were below the 10^th^ percentile and above the 90^th^ percentile of the average weight of the same gestational age, with the 2015 Chinese neonatal birth weight curve for different gestational ages used as reference [19]. **Macrosomia**: the birth weight of the newborn ≥ 4,000 g. **Pre-pregnancy BMI category**: underweight (< 18.5 kg/m^2^), normal (18.5–23.9 kg/m^2^), overweight (24.0–27.9 kg/m^2^) and obesity (≥ 28.0 kg/m^2^) ( according to the “Guidelines for prevention and control of overweight and obesity in China”) [20]. **Excessive GWG**: underweight ≥ 17.1 kg, normal ≥ 16.4 kg, overweight/ obesity ≥ 14.9 kg [21].

### Laboratory methods

Peripheral venous blood was drawn during the first trimester (6–12 weeks of pregnancy) and the second trimester (24–28 weeks of pregnancy), after the GDM women had fasted for 8–12 h over night. The blood sample was centrifuged at 3,000 rpm for 10 min to obtain serum for analysis. In the first trimester, all samples were tested for fasting plasma glucose (FPG), HbA1c, total cholesterol (TC), TG, total protein (TP), and albumin (ALB) levels. All samples from 24–28 weeks of gestation were tested for serum fasting insulin, fasting plasma glucose, and plasma glucose level both 1 h and 2 h after orally taking glucose. Fasting insulin was tested via electrochemiluminescence immunoassay (ROCHE COBASE 601), the analytical sensitivity was 0.2μU/mL, the coefficient of variation was 1.5%. HbA1c was tested by HPLC (Premier Hb9210), the analytical sensitivity was 3.8%, the coefficient of variation was < 1.5%. Blood glucose was tested by the hexokinase method (When the concentration of the reagent is about 8.0 mmol/L, the absorbance range is 0.30–0.45, the coefficient of variation was ≤ 3%), TC was tested by the peroxidase method (When the concentration of the reagent is about 4.9 mmol/L, the absorbance range is 0.16–0.31, the coefficient of variation was ≤ 3%), TG was tested by the coupling-POD enzyme colorimetric method (When the concentration of the reagent is about 1.85 mmol/L, the absorbance range is 0.097–0.200, the coefficient of variation was ≤ 5%), TP by the biuret method (When the concentration of the reagent is about 70 g/L, the absorbance range is 0.35–0.60, the coefficient of variation was ≤ 3%), and ALB by bromomethylolate green (When the concentration of the reagent is about 42 g/L, the absorbance range is 0.50–0.75, the coefficient of variation was ≤ 2%). All the tests were performed in an automatic biochemical analyzer (BECKMAN AU5821).

The homeostasis model assessment (HOMA) was used to evaluate IR and β-cell function in GDM. HOMA-IR = fasting plasma insulin concentration (μU/mL) × fasting plasma glucose (mmol/L) / 22.5. HOMA-β = 20 × fasting plasma insulin concentration (μU/mL) / (fasting plasma glucose [mmol/L]-3.5). According to the HOMA value, the cut-off value was set at 2.0, and then the patients were divided into the non-IR GDM group (HOMA < 2) and IR GDM group (HOMA-IR ≥ 2) [22].

### Statistical analysis

SPSS 23.0 was used for statistical analysis. All variables were fully observed for all participants. The counting data were expressed as frequency and rate (%), and Chi-square test was adopted. The Shapiro–Wilk test was used to determine the data normality. For continuous variable data in a normal distribution, the mean and standard deviation were chosen for description, and the t-test for comparison between groups. For the continuous variable data in a skewed distribution, the median and quartile were chosen, and the Mann–Whitney U test was used for comparison between groups. The multivariable logistic regression model was performed to measure the potential risk factors of IR by calculating the odds ratio (OR) and 95% confidence interval (CI). The univariable and multivariable logistic regression models were used to analyze the relationship between IR and adverse pregnancy outcomes.

## Results

### General characteristics of the mothers and newborns among the compared groups

Seven hundred ten GDM women were included, aging from 23 to 51 years old with an average age of 33.00 ± 4.18 years old. All of them made regular medical visits to the Obstetrics Clinic of the Sixth Medical Center of PLA General Hospital. Their gestational week of delivery ranged from 32 to 42 weeks, with an average of 38.13 ± 1.49 weeks.

Table 1 shows the clinical characteristics of GDM women and their newborns, and there were no difference in the age, parity, history of GDM, family history of diabetes, IVF-ET, GWG and weight gain before diagnosis of GDM between the two groups. Stratified by pre-pregnant BMI, weight distribution ( underweight, normal, overweight and obesity) was significantly different in the two groups (*p* < 0.001). Women in the IR GDM group had worse glycemic control (*P* = 0.04), and were more likely to be given insulin therapy. 698 women controlled the blood sugar by diet and exercise, however, only 12 women received insulin therapy based on diet and exercise, and all of them were from the IR GDM group. For metabolic parameters, the FPG (*P* < 0.001), HbA1c (*P* < 0.001), TC (*P* = 0.003), and TG (*P* < 0.001) of IR GDM women during early pregnancy were significantly higher than the non-IR GDM group. However, TP and ALB were similar between the two groups (*P* = 0.061, *P* = 0.835). Pancreatic β-cell function in the IR GDM group was significantly enhanced (*P* < 0.001). Although the GWG and weight gain before diagnosis of GDM were no statistically significant differences between the two groups, as for excessive GWG, IR GDM group increased significantly than the non-IR GDM group (*P* < 0.001). In addition, with 30 premature birth cases excluded, there was also no significant difference in GWG (*P* = 0.638), but there was a statistically significant difference in the birth weight of the newborns between the two groups (*P* = 0.014).

**Table 1:** General characteristics comparison

	Non-IR GDM	IR GDM	Test Statistic	*P*
**Number**	418	292		
**Age (years)**	33.20 ± 4.127	32.72 ± 4.255	1.495	0.135
**Parity**			1.014	0.314
Primiparity	239(57.3%)	178(42.7%)		
Multiparity	179(61.1%)	114(38.9%)		
**Glycemic control**			6.441	0.040
Good	412(59.5%)	280(40.5%)		
Poor	6(33.3%)	12(66.7%)		
**IVF-ET**			0.268	0.604
Yes	48(61.5%)	30(38.5%)		
No	370(58.5%)	262(41.5%)		
**History of GDM**			0.669	0.413
Yes	10(50%)	10(50%)		
No	408(59.1%)	282(40.9%)		
**Family history of diabetes**			2.190	0.139
Yes	98(54.1%)	83(45.9%)		
No	320(60.5%)	209(39.5)		
**Pre-pregnant BMI category**			97.157	< 0.001
Underweight (less than18.5)	51(89.5%)	6(10.5%)		
Normal (18.5 to 23.9)	307(66.6%)	154(33.4%)		
Overweight (24.0 to 27.9)	50(35.2%)	92(64.8%)		
Obesity (28 or more)	10(20%)	40(80%)		
**FPG (mmol/L)**	4.70 ± 0.44	4.85 ± 0.49	-4.145	< 0.001
**HbA1c (%)**	5.26 ± 0.28	5.38 ± 0.30	-5.684	< 0.001
**HOMA-β**	85.64(68.94,110.67)	141.36(114.13,182.18)	18,787.000	< 0.001
**Serum Lipid**				
**TC (mmol/L)**	4.35 ± 0.83	4.55 ± 0.93	-2.985	0.003
**TG (mmol/L)**	1.23 ± 0.92	1.59 ± 1.46	-3.792	< 0.001
**Serum protein**				
**TP (mmol/L)**	73.0 ± 4.13	73.57 ± 3.90	-1.873	0.061
**ALB (mmol/L)**	42.53 ± 3.15	42.48 ± 2.83	0.209	0.835
**Weight gain before diagnosis of GDM (kg)**	5.24 ± 3.00	5.36 ± 3.27	-0.506	0.613
**GWG (kg)**	10.65 ± 4.57	10.96 ± 5.40	-0.893	0.402
**Excessive GWG**			32.949	< 0.001
Yes	41(35%)	76(65%)		
No	377(63.6%)	216(36.4%)		
^**a**^**GWG (kg)**	10.77 ± 4.53	10.96 ± 5.34	-0.47	0.638
^**a**^**Birth weight (g)**	3343.60 ± 390.24	3423.77 ± 434.55	-2.461	0.014

^a^ Non-IR GDM group (n 404), IR GDM group (n 276), with 30 cases of preterm birth excluded; *IVF-ET* In vitro fertilisation-embryo transfer; Test Statistic: *P*-values were estimated from the resulting *t*-values for normally distributed data, and were estimated from the resulting *U*-values for non-normally distributed data, and were estimated from the resulting *χ2*-values for counting data

### Risk factors of IR

Meanwhile, the risk factors for IR were explored. Multiple stepwise regression analyses were performed with independent variables including age, pre-pregnancy BMI, FPG, HbA1c, TP, ALB, TC, TG, parity, history of GDM, family history of diabetes, IVF-ET, weight gain before diagnosis of GDM. It can be seen from the data in Table 2, the statistically significant factors of IR were pre-pregnancy BMI (*P* < 0.001), FPG (*OR* = 1.63, *95%CI* 1.11, 2.40), TG (*OR* = 1.32, *95%CI* 1.08, 1.63) and weight gain before diagnosis of GDM (*OR* = 1.08, *95%CI* 1.02, 1.15). According to BMI stratification analyses, the risks of GDM with IR rose with the greater of pre-pregnancy BMI category: the ORs (95%CI) of underweight, overweight and obesity were 0.20(0.08, 0.50), 4.09 (2.65, 6.30), and 6.52 (2.99, 14.20) when compared with the normal group. However, age was the weak protective factor for GDM with IR (*OR* 0.94, *95%CI* 0.90, 0.98).

**Table 2:** Multiple stepwise regression analysis between HOMA-IR and various indicators

Variable	*OR(95%CI)*	*P*
Age (years)	0.94(0.90, 0.98)	0.003
Pre-pregnant BMI category		< 0.001
Underweight (less than18.5)	0.20(0.08, 0.50)	0.001
Normal (18.5 to 23.9)	1.00	
Overweight (24.0 to 27.9)	4.09 (2.65, 6.30)	< 0.001
Obesity (28 or more)	6.52 (2.99, 14.20)	< 0.001
TG (mmol/L)	1.32 (1.08, 1.63)	0.008
FPG (mmol/L)	1.63 (1.11, 2.40)	0.013
HbA1c (%)	3.11 (1.68, 5.73)	< 0.001
Weight gain before diagnosis of GDM (kg)	1.08 (1.02,1.15)	0.007

Variables including age, pre-pregnancy BMI category, FPG, HbA1c, TP, ALB, TC, TG, parity, history of GDM, family history of diabetes, IVF-ET, Weight gain before diagnosis of GDM

The reported ORs are unadjusted for the other variables which remained in the model

### The relationship between IR and adverse pregnancy outcomes

IR significantly increased the risk of the hypertensive disorders of pregnancy and LGA (*OR* = 5.31,*95%CI*:1.87,15.10; *OR* = 1.65,*95%CI*:1.10, 2.48, respectively) in women with GDM, but not for cesarean, premature delivery, premature rupture of membranes, postpartum hemorrhage, macrosomia and SGA. In addition, multiparity (*OR* = 1.89,*95%CI*:1.24–2.86) and increasing GWG (*OR* = 1.06,*95%CI*: 1.01–1.12) also significantly increased the risk of LGA in women with GDM (Table 3).

**Table 3:** Influence of HOMA-IR on pregnancy outcome of GDM pregnant women

	Univariable logistic regression		Multivariable logistic regression	
	*OR(95%CI)*	*P*	*OR(95%CI)*	*P*
Cesarean section	1.26 (0.92,1.72)	0.143	1.13 (0.78, 1.65)	0.517
Premature delivery	1.80 (0.85, 3.81)	0.123	1.94 (0.80, 4.74)	0.146
Premature rupture of membranes	1.32 (0.93, 1.86)	0.120	1.03 (0.69, 1.53)	0.897
Postpartum hemorrhage	1.12 (0.72, 1.74)	0.607	1.03 (0.62, 1.72)	0.905
Hypertensive disorders of pregnancy	8.40 (3.19, 22.07)	< 0.001	5.31 (1.87, 15.10)	0.002
Macrosomia	1.62 (0.87, 3.03)	0.130	0.97 (0.46, 2.07)	0.940
LGA	2.04 (1.44, 2.89)	< 0.001	1.65 (1.10, 2.48)	0.016
SGA	1.56 (0.74, 3.29)	0.24	1.41 (0.55, 3.61)	0.477

Confounding factors:age, parity, IVF-ET, pre-pregnancy BMI category, FPG, HbA1c, TC, TG, TP, ALB, excessive GWG, GWG, glycemic control

### The relationship between glycemic control/excessive GWG and adverse pregnancy outcomes

By univariable logistic regression analysis, poor glycemic control (OR = 1.26, *95%CI:*0.92,1.72) significantly increased the risk of cesarean, compared to good glycemic control (Table 4). And excessive GWG significantly increased the risk of hypertensive disorders of pregnancy (*OR* = 3.44, *95%CI*:1.62,7.29), macrosomia (*OR* = 3.85, *95%CI*:2.00,7.39) and LGA (*OR* = 2.58,*95%CI:*1.70,3.92) (Table 5). However, after controlling for confounding factors, no association between glycemic control/excessive GWG and adverse pregnancy outcomes was revealed (P > 0.05) (Tables 4 and 5).

**Table 4:** Influence of glycaemic control on pregnancy outcome of GDM pregnant women

	Univariable logistic regression		Multivariable logistic regression	
	*OR(95%CI)*	*P*	*OR(95%CI)*	*P*
Cesarean section	2.97 (1.14, 7.77)	0.026	2.15 (0.71, 6.52)	0.178
Premature delivery	3.07 (0.67, 14.03)	0.148	4.26 (0.84, 21.75)	0.081
Premature rupture of membranes	1.22 (0.43, 3.48)	0.708	1.43 (0.47, 4.32)	0.529
Postpartum hemorrhage	1.91 (0.61, 5.92)	0.265	1.41 (0.41, 4.89)	0.589
Hypertensive disorders of pregnancy	1.25 (0.16, 9.70)	0.831	0.77 (0.85, 6.99)	0.818
Macrosomia	2.03 (0.45, 9.16)	0.355	1.07 (0.19, 5.93)	0.935
LGA	1.25 (0.44, 3.55)	0.680	0.69 (0.21, 2.24)	0.538
SGA	0.00 (0.00, /)	0.998	0.00 (0.00, /)	0.998

Confounding factors:age, parity, IVF-ET, pre-pregnancy BMI category, FPG, HbA1c, TC, TG, TP, ALB, excessive GWG, GWG, HOMA-IR

**Table 5:** Influence of excessive GWG on pregnancy outcome of GDM pregnant women

	Univariable logistic regression		Multivariable logistic regression	
	*OR(95%CI)*	*P*	*OR(95%CI)*	*P*
Cesarean section	1.35 (0.90, 2.02)	0.147	0.80 (0.43, 1.47)	0.472
Premature delivery	0.84 (0.28, 2.45)	0.743	1.72 (0.40, 7.43)	0.471
Premature rupture of membranes	1.28 (0.82, 2.01)	0.272	1.16 (0.61, 2.22)	0.645
Postpartum hemorrhage	1.44 (0.84, 2.47)	0.187	0.97 (0.44, 2.17)	0.943
Hypertensive disorders of pregnancy	3.44 (1.62, 7.29)	0.001	1.04 (0.31, 3.55)	0.945
Macrosomia	3.85 (2.00, 7.39)	< 0.001	2.08 (0.72, 6.03)	0.178
LGA	2.58 (1.70, 3.92)	< 0.001	1.35 (0.72, 2.54)	0.351
SGA	1.10 (0.41, 2.96)	0.85	1.20 (0.25, 5.69)	0.82

Confounding factors:age, parity, IVF-ET, pre-pregnancy BMI category, FPG, HbA1c, TC, TG, TP, ALB, GWG, HOMA-IR, glycemic control

## Discussion

### IR harms the prognosis of GDM women

From the results of this retrospective study, IR was found to be harmful to the GDM’s prognosis, with an increase in adverse outcomes. When the confounding factors were excluded, the incidences of hypertensive disorders of pregnancy and LGA were significantly higher.

#### GDM with IR and hypertensive disorders of pregnancy

In our study, compared with the GDM women without IR, the risk of hypertensive disorders of pregnancy increased up to 5 times in GDM women with IR. An Iranian study [23] had similar findings: insulin resistance, closely related to preeclampsia, served as a risk factor in the process. Indeed, before clinical symptoms occur, the fasting insulin level increases accordingly as the disease progresses. Based on this, identifying GDM women with IR could be considered as a starting point for the clinical prevention of hypertensive disorders of pregnancy. However, the specific mechanism is still unclear. In the mainstream opinion, physiological IR provides more glucose to the fetus to better nourish its development, but overactivation of IR in pregnancy leads to decreased carbon monoxide (NO), discorded lipid metabolism, low prostaglandin E2 synthesis, and damaged vascular endothelial cells, eventually resulting in maternal high blood pressure [24, 25].

#### GDM with IR and LGA

GDM with IR may also cause fetal overgrowth. In our study, babies born to GDM women with IR had a significant increase in average birth weight compared with the babies of GDM women without IR. With the confounding factors (age, BMI, TG, FPG, HbA1c, etc.) adjusted, IR was found to be an independent risk factor of LGA, doubling the risk. This discovery is similar to that of Tressa Ellett’s research [26], in which IR was positively correlated to the birth weights of newborns. The mechanism of this phenomenon has been partially explained. In Pedersen's [27] hypothesis, maternal hyperglycemia passing through the placental barrier stimulates the fetus to secrete more insulin which promotes fat and protein accumulation, leading to excessive fetal growth. As a result, high-level maternal insulin, which though cannot go through the placenta, causes changes in the placenta’s metabolism, gene expression and epigenetic modifications [28], eventually disturbing fetal growth.

#### Glycaemic control/excessive GWG and adverse pregnancy outcomes

Our findings were inconsistent with the results of previous studies [29, 30]. In our study, poor glycemic control and excessive GWG increased the risk of hypertensive disorders of pregnancy, macrosomia, LGA and cesarean by univariable logistic regression analysis, but not by multivariable logistic regression analysis. Analysing the reasons of the abovementioned result, it was possible to consider that the sample size in certain subgroups was relatively small, so it leaded to a larger span of 95% CI. In addition, the inconsistent diagnosis criteria was another possible reason, that we employed criteria of pre-pregnancy BMI category [20] and excessive GWG [21] which were more suitable for Chinese people.

### IR’s risk factors

Previous research has mainly focused on the risk factors of IR or GDM, but few referred to the case of GDM with IR, which we analyzed in our study. Based on our data, TG, HbA1c, and FPG in early pregnancy, pre-pregnant BMI, weight gain before diagnosis of GDM were considered to be the risk factors of GDM with IR. Our results are similar to the results of Sun’s study [11], in which BMI and TG were related to GDM with IR in the second trimester, but FPG and HbA1c were not. The differences in the results possibly derive from the fact that our FPG and HbA1c were tested in early pregnancy, while Sun’s results were obtained from the second trimester. Meanwhile, it is worth noting that FPG and HbA1c, although within normal range, were higher in GDM women with IR than that in those without IR, which indicates that more benefit will occur if blood sugar control is performed early. However, considering the dangers of hypoglycemia, more research needs to be done to determine the appropriate control range.

In addition, we found that weight gain before diagnosis of GDM was another risk factor of GDM with IR in the second trimester, which was consistent with literature reports. A retrospective study was conducted among 2,647 women with GDM which showed a significantly increased risk of IR for women with more weight gain before diagnosis of GDM [11]. Thus, in future clinical work, we should enhance weight management initiating from the first trimester for high-risk women to improve insulin resistance.

Furthermore, in our research, age was a weak protective factor for IR. However, Sun reached the opposite conclusion [11]. We consider that this difference in results comes from the fact that participants from Sun’ study were divided into four groups according to the severity of insulin resistance, defined by quartiles of HOMA-IR (1.540, 2.269, and 3.238), however, divided into two groups by HOMA-IR (2.0) for us. Therefore, uniform grouping criteria and expanding the study population are needed to confirm the results.

### Management of GDM with IR

In this study, it was found that the HOMA-β value is increased in GDM women with IR. which is considered that IR promotes pancreatic β-cells to produce and secrete more insulin. This compensatory hyperinsulinemic response of β-cells can initially control blood glucose within the normal range; however, long-term exposure to excessive glucose and lipids will eventually lead to β-cell dysfunction and / or cell death, leading to overt diabetes. The protection of pancreatic β-cell function is closely related to the reduction of endogenous insulin demand [31]. Therefore, women with insulin resistance to GDM are likely to benefit from insulin sensitization therapy, providing a new approach for the individualized treatment of GDM. This view is similar to that of a study in Poland, in which Sokup [32] suggested that GDM women with HOMA > 1.29 should be treated with improved tissue sensitivity to insulin, while GDM women with HOMA > 2.89 should be treated with metformin combined with insulin.

It is worth noting that the current clinical management goal of GDM is blood glucose. In fact, a high blood glucose level can only explain a small part of the variation in birth weight [33]. In this study, although the most of women with GDM had a normal blood glucose range, women with GDM with insulin resistance had a higher risk of LGA. Our conclusion is consist with Li’s study [34]. Therefore, insulin resistance should also be an important indicator in pregnancy monitoring, especially for GDM women within the normal range of blood glucose and with high risk factors.

In particular, in this study, only 1.7% of the GDM women were treated with insulin therapy to control blood glucose, which is quite different from in other countries. In Australia, this proportion can reach to 36.8% [35]. This difference is considered to result from the lack of consensus and consistency in the screening and diagnostic criteria for gestational diabetes in various countries. In China, the diagnosis of GDM is up to the more rigid IADPSG standard, in which the cut-off value is lower than in other guidelines. The IADPSG criteria increase the prevalence of GDM [36]. However, alternative standards are still used in some centers and countries including Australia [37]. This has led to the expansion of the GDM population in China, while the proportion of women who really need insulin treatment has decreased. Therefore, it is worth exploring whether the IADPSG standard is applicable to the Asian population. We further suggest an appropriate stratification of the management of GDM women based on HOMA-IR to reduce the loss of medical resources. In addition, there are still 18 women in this study with poor glycemic control, due to various reasons (because of the retrospective study, the exact reason cannot be obtained), did not use insulin therapy, which was also one of the reasons for the low rate of insulin use in this study.

### Limitations

First, this study was a retrospective study, in which bias was inevitably introduced in the participant selection. Second, our research subjects were GDM pregnant women, so the results would not be applicable to non-GDM women. Third, our model did not include other risk factors that affect IR, including diet, physical activity, smoking and drinking. In addition, HOMA-IR before childbirth was not tested, so it was not clear whether the women’s IR was improved by lifestyle and diet management. In the end, more research is needed to dynamically observe the changes in IR before pregnancy, during pregnancy, and after delivery, and to clarify the influencing factors of IR and the relationship between IR and GDM.

## Conclusion

In summary, our research shows that IR in the second trimester is associated with adverse pregnancy outcomes such as hypertensive disorders of pregnancy and LGA. Moreover, TG, FPG, and HbA1c in the first trimester, pre-pregnancy BMI and weight gain before diagnosis of GDM are independent risk factors for IR in the second trimester.

## Data Availability

The dataset used in the present study is available from the corresponding author upon reasonable request.

## Abbreviations

IR: Insulin resistance
GDM: Gestational diabetes mellitus
FPG: Fasting plasma glucose
HbA1c: Glycosylated hemoglobin
TC: Serum total cholesterol
TG: Serum total triglyceride
TP: Serum total protein
ALB: Serum albumin
BMI: Body mass index
SGA: Small for gestational age infant
LGA: Large for gestational age infant
HOMA-IR: Homoeostasis model assessment of IR
HOMA-β: Homoeostasis model assessment of β-cell function
GWG: Total weight gain during pregnancy
OGTT: 75G oral glucose tolerance test
IVF-ET: In vitro fertilization -embryo transfer
